# Barriers and facilitators to symptom-related help-seeking among men with genitourinary malignancies: a qualitative study

**DOI:** 10.3389/fonc.2026.1915743

**Published:** 2026-10-09

**Authors:** Rong Liu, Xiang Xi, Xiao Ma, Yu Zhao, Hui Zhuo

**Affiliations:** Chengdu Third People’s Hospital, Chengdu, China

**Keywords:** genitourinary malignancies, psychosocial factors, qualitative research, symptom-related help-seeking, symptom management

## Abstract

**Objective:**

To explore perceived barriers and facilitating factors related to symptom-related help-seeking among men with genitourinary malignancies and inform the development of symptom-management support.

**Methods:**

Men with genitourinary malignancies were recruited through purposive sampling at a tertiary hospital. Data were collected through semi-structured, in-depth interviews and analyzed using Colaizzi’s seven-step method.

**Results:**

Fifteen men were interviewed, and two themes with five subthemes were identified. Barrier factors comprised limited symptom knowledge, psychosocial concerns, and difficulties accessing information and symptom-management support. Facilitating factors comprised social and family support and perceived benefits of seeking professional help.

**Conclusions:**

The findings suggest potential priorities for symptom-management support, including symptom education, family and social support, and accessible professional advice. Their relevance should be considered in relation to the cancer types, age distribution, and care setting represented in this study.

## Introduction

Male genitourinary malignancies include cancers of the kidney, prostate, bladder, testis, ureter, and penis (1–4). Cancer statistics in the United States indicate that the highest prevalence of cancer in men is prostate cancer, followed by bladder, kidney, and testicular cancer (5, 6). The increase in its incidence may be related to factors such as population aging, modern lifestyle changes, increased exposure to risk factors, advances in disease screening and diagnosis, and improved residents’ awareness of self-protection (7). Early cancer diagnosis and treatment are key to effective and efficient care, and one factor associated with timely access to treatment is seeking medical help for potential cancer symptoms (8, 9).

Symptom-related help-seeking refers to decisions about seeking medical help and using healthcare services after noticing a potential cancer symptom. Factors discussed in the literature include age, marital status, educational level, family history of cancer, fear of cancer, perceived threat, and disclosure of symptoms to significant others. In the Chinese cultural context, expectations concerning men’s family and social roles may shape their experiences of illness (10). For cancers involving the urinary tract or male reproductive organs, concerns about intimate symptoms and sexual function may make it difficult for men to seek medical help and discuss their needs (11).

Research has examined help-seeking before cancer diagnosis, but further work is needed to understand how men with different genitourinary malignancies describe help-seeking across diagnosis and treatment (12, 13). The Health Belief Model (HBM) offers concepts for interpreting perceived threat, benefits, barriers, cues to action, and self-efficacy in health behavior (14, 15). Its relevance to the present findings is considered in the Discussion.

This descriptive phenomenological study explored men’s experiences of symptom-related help-seeking in the context of genitourinary malignancies. It aimed to identify perceived barriers and facilitators and inform priorities for nursing support.

## Methods

### Informants

From July to December 2025, men with genitourinary malignancies who returned for treatment at the Department of Urology, the Third People’s Hospital of Chengdu, China, were recruited through purposive sampling. The stated inclusion criteria were male sex, age ≥18 years, a genitourinary malignancy, receipt of surgery, radiotherapy, or chemotherapy at the hospital, awareness of the diagnosis, and willingness to participate. The stated exclusion criteria were severe mental illness, cognitive impairment, communication difficulties, other cancers, or participation in similar studies. Fifteen informants were included. No new themes were reported during analysis of the overall interview material (16); this does not establish data adequacy within individual cancer types or age groups. The study was approved by the Ethics Committee of the Third People’s Hospital of Chengdu (No. 2025-S-3).

### Development of the interview guide

The research team developed a semi-structured interview guide based on the literature and clinical experience. Pilot interviews were conducted with one informant with bladder cancer and one with prostate cancer. The team refined the guide using feedback from these informants and six experts: three chief physicians in urological oncology, two clinical nursing experts, and one psychologist. The interviewer clarified questions when informants had difficulty understanding them. The nine questions are presented in the interview guide below (see **Table 1**).

**Table 1:** Interview outline.

Interview guide
1. From the time of diagnosis to the present, what symptoms have you noticed that you associate with your genitourinary cancer?
2. How do you perceive these symptoms?
3. What aspects of these symptoms concern you?
4. How have you responded to these symptoms or sought help?
5. What difficulties have you encountered?
6. What factors have hindered or facilitated your help-seeking for symptoms related to genitourinary cancer?
7. What other needs do you have regarding symptom-related help-seeking and symptom management?
8. What kind of help would you like? 9. Is there anything else you would like to add?

### Data collection and quality control methods

Individual interviews were conducted in a quiet consultation room, with only the interviewer and informant present, and lasted 60–90 minutes. The interviewer was a nursing team leader with clinical experience in caring for men with genitourinary malignancies. Before each interview, the study purpose and procedures were explained, confidentiality was discussed, and written informed consent was obtained. Interviews were audio-recorded, and field notes documented facial expressions, body movements, and recurrent expressions. Recordings were transcribed and checked with the informants for accuracy.

### Data analysis methods

Interview data were analyzed using Colaizzi’s method (17). Recordings were transcribed after the interviews and checked against field notes. Team members trained in qualitative research, including a nursing graduate student, repeatedly read the transcripts and identified statements relevant to symptom-related help-seeking. Two analysts developed preliminary interpretations of these statements and formulated meaning units. NVivo was used to organize the coded material. Differences in coding and theme development were discussed within the research team. Transcripts were checked with informants for accuracy.

## Results

Fifteen informants, identified as I1–I15, were aged 28–81 years(61.73 ± 14.58). **Table 2** summarizes their characteristics. The sample included four informants with bladder cancer, four with prostate cancer, three with kidney cancer, two with penile cancer, and two with testicular cancer. Eleven informants were married, three were widowed, and one was unmarried. Two themes and five subthemes were identified (**Table 3**).

**Table 2:** General information of the study subjects.

Informant number	Age	Marital status	Educational level	Employment status	Place of residence	Type of tumor	Tumor clinical staging	Treatment
I1	28	Unmarried	Junior high school and below	Employed	Urban	Penile cancer	Stage I	Surgical treatment
I2	66	Married	High school	Unemployed	Rural	Bladder cancer	Stage III	Surgical treatment
I3	53	Married	High school	Unemployed	Urban	Kidney cancer	Stage IV	Targeted therapy combined with immunotherapy
I4	38	Married	College or above	Employed	Urban	Testicular cancer	Stage II	Surgical treatment
I5	52	Married	High school	Employed	Urban	Kidney cancer	Stage III	Targeted Therapy
I6	64	Married	College or above	Retired	Urban	Bladder cancer	Stage II	Surgical treatment
I7	81	Widowed	Junior high school and below	Retired	Rural	Prostate cancer	Stage III	Surgical treatment
I8	74	Married	College or above	Retired	Urban	Prostate cancer	Stage II	Surgical treatment
I9	67	Married	College or above	Retired	Urban	Prostate cancer	Stage II	Surgical treatment
I10	67	Widowed	College or above	Retired	Urban	Bladder cancer	Stage II	Surgical treatment
I11	67	Married	Junior high school and below	Retired	Rural	Kidney cancer	Stage III	Surgical treatment
I12	75	Married	Junior high school and below	Unemployed	Rural	Prostate cancer	Stage IV	Targeted Therapy
I13	75	Widowed	Junior high school and below	Unemployed	Urban	Bladder cancer	Stage IV	Targeted therapy combined with immunotherapy
I14	52	Married	High school	Employed	Urban	Penile cancer	Stage III	Surgical treatment
I15	67	Married	Junior high school and below	Retired	Rural	Testicular cancer	Stage IV	Targeted Therapy

**Table 3:** Themes, subthemes, and categories.

Themes	Subthemes	Categories
Barrier factors	Limited symptom knowledge and risk awareness	Mild symptoms and benign explanations
		Trivialization of symptoms
	Psychosocial barriers to symptom-related help-seeking	Sexual function concerns and stigma
		Negative emotions and symptom concealment
		Family responsibilities and gender role expectations
	Limited access to information and symptom-management support	—
Facilitating factors	Social and family support for symptom-related help-seeking	—
	Perceived benefits of seeking professional help	—

### Theme 1: barrier factors

#### Subtheme 1: limited symptom knowledge and risk awareness

This subtheme describes how informants interpreted symptoms and judged whether medical attention was needed. Their accounts reflected two patterns: attributing symptoms to benign causes and minimizing the importance of symptoms.

##### Mild symptoms and benign explanations

Informants attributed symptoms to gastrointestinal problems, prostatic enlargement, or reduced immunity. These explanations appeared to make the symptoms less concerning and reduced their perceived need for assessment.

I2: “Before I was diagnosed, I had a dull pain in my lower abdomen, but I thought it was a gastrointestinal problem. The pain was not very noticeable, so I thought it was nothing to worry about.”

I3: “I had been going to the toilet frequently. At night, I had to get up about five or six times. I thought there was something wrong with my bladder or kidneys; I did not know it was cancer.”

I10: “I often get up at night to go to the toilet, and I feel that I have not finished urinating. I think it is an enlarged prostate, so I buy medicine and take it myself.”

I4: “Since last year, I have often had colds and fevers, and I have been losing weight. I thought my immune system had become weaker.”

##### Trivialization of symptoms

Informants described symptoms as familiar, mild, or insufficiently disruptive to warrant medical attention. Some interpreted urinary changes as part of aging, whereas others focused on another health problem. These accounts suggest that noticing a bodily change did not necessarily lead to recognizing a need for further assessment.

I1: “What did I know about cancer symptoms? I thought frequent urination and pain when urinating were normal as you got older.”

I2: “I noticed changes in my urination, but I thought they were minor because they happened almost every day and did not seriously affect my life.”

I9: “I had been healthy, but later I found out that my blood pressure was high. I was treated for high blood pressure, and a mass was found during a medical examination.”

#### Subtheme 2: psychosocial barriers to symptom-related help-seeking

##### Sexual function concerns and stigma

This subtheme concerns emotional and social pressures that made symptom disclosure or help-seeking difficult. Informants described embarrassment about sexual concerns, fears about treatment and illness, and tensions between seeking care and fulfilling family or masculine roles. The following accounts illustrate concerns about sexual function and anticipated judgment by others.

I11: “I’m embarrassed to say that I have a problem with that (bladder); people may think that I did not go out to have sex before. As a man, people will laugh at me if I lose my basic male function…” I4: “The doctor told me that the operation would involve removing part of the gland. I am afraid this will affect my sexual function and our sex life as a couple. I also feel embarrassed discussing sex with the healthcare staff, my family, or my friends.”

I14: “It has been a long time since the operation, but I still do not dare to look at my penis. Part of it was removed. I feel that I have lost part of what makes me a man. I am afraid that the operation may not have cured the disease. What is the point of being a man now? I feel as though I can no longer hold my head up.”

##### Negative emotions and symptom concealment

Informants described fear, discouragement, and reluctance to discuss illness or symptoms. Some concealed symptoms to avoid worrying family members, whereas others doubted whether professionals could help or were reluctant to undergo further treatment. These accounts illustrate different reasons for withholding concerns rather than a single emotional response shared by all informants.

I14: “When I noticed that my urine was red, I felt frightened and panicked. I did not tell my family or friends because I did not want them to feel the same distress. I monitored my symptoms and bought medicine myself. After several days without improvement, I went to hospital.”

I6: “I do not think there is anything more the doctors can do. They are doing everything they can. I do not have much confidence; I will die sooner or later.”

I5: “I’m scared. Because I don’t go very often, I don’t like to talk about things…… Because a year ago, a few years ago, I had a lot of surgery, and I just didn’t want to do it again.”

I2: “I noticed changes in my body at first, but I could not find anyone to talk to. When I was diagnosed, I tried to forget that I had cancer. I wanted to act as though it did not exist and avoid conversations about cancer. I wanted my life to go back to how it was before.”

I15: “I thought the symptoms were minor. Going to the doctor or nurse every day might bother them because they were already busy. Going to hospital every day also put a strain on my family and made me feel increasingly useless.”

##### Family responsibilities and gender role expectations

This category describes tensions between seeking care and meeting work, family, or gender-related expectations. Some informants prioritized work or feared that taking leave would threaten their ability to support their families. Others felt that enduring symptoms was preferable to being judged as making a fuss.

I15: “At first, I did not think it was very serious. Treatment is uncomfortable, so I thought I should not worry about every small problem. I was afraid people would think I was making a fuss or talk about me behind my back. I thought the symptoms would go away after a while and that I could cope with them.”

I7: “I know I have symptoms, but I am too busy with work to see a doctor. I have to finish my work before I can think about my health.”

I4: “I have older family members and children to support. Taking too much medical leave will affect my work. If I lose my job because of this illness and these symptoms, what will my family do?”

#### Subtheme 3: limited access to information and symptom-management support

Due to limited knowledge of the disease and limited resources for home rehabilitation, most patients hoped to learn more about the disease from medical staff, clarify disease progression, and understand subsequent treatment and follow-up plans. Their accounts suggested that information needs were not always met during clinical encounters. Informants also wanted supportive communication and professional guidance on rehabilitation and symptom management.

I8: “I want the doctor to explain my illness and treatment clearly. The hospital is far from my home. If I develop symptoms after going home, I hope I can contact doctors or nurses online for professional advice.”

I9: “The internet is becoming more widely used, but it is still difficult for older people like me. I have a stoma on my abdomen, but I struggle to care for it and change the bag myself. I wish the hospital and community health center offered regular education and professional help.”

I11: “I know cancer is difficult to cure, but I have also had side effects during treatment and have been to hospital several times this year. I wish the staff had explained the treatment, its side effects, and what to look out for before treatment started.”

### Theme 2: facilitating factors

#### Subtheme 1: social and family support for symptom-related help-seeking

This subtheme describes how family members, healthcare staff, and other patients supported symptom disclosure and engagement with care. Family encouragement prompted one informant to seek assessment, while reassurance and practical encouragement helped another cope after surgery. These examples relate to different points in the care trajectory.

I13: “My urine was red for several days. I was terrified and told my family. They insisted that I go to the hospital to have it checked.”

I7: “In the first few days after surgery, I felt low. My wound was very painful, and I had severe abdominal bloating. I felt anxious and sometimes cried privately. I appreciated my wife, the healthcare staff, and other patients encouraging me to relax and get out of bed. As my condition improved, I became more willing to engage with treatment. I am now about to be discharged. The doctor advised regular follow-up, and I plan to monitor changes in my body.”

#### Subtheme 2: perceived benefits of seeking professional help

This subtheme describes how informants experienced professional advice as useful and reassuring. Access to information and responsive communication made support valuable during symptom management. The accounts show perceived benefits of seeking help, but do not establish that these experiences changed later help-seeking behavior.

I8: “I joined the department’s WeChat group after discharge, and the staff regularly shared information about the disease. I felt it was very useful. When I encountered problems with the symptoms, I would also ask related questions in the group, and the medical staff would patiently answer my questions.”

I13: “After the catheter was removed, I could not pass urine and my abdomen became very swollen, so it was put back in. I was terrified that it would never be removed. I am grateful that the staff explained what had happened, listened to my concerns, and helped me prepare for catheter removal. That made me less afraid. The catheter was eventually removed successfully.”

## Discussion

This study identified individual, interpersonal, and service-related considerations in symptom-related help-seeking among 15 men with genitourinary malignancies. The accounts suggest that recognizing a symptom, feeling able to disclose it, and obtaining professional advice are related but distinct challenges. They also span prediagnostic symptom appraisal and symptom management during treatment. The contribution is therefore an account of perceived needs across these experiences, rather than evidence of a uniform pathway shared by all cancer types.

Symptom recognition did not necessarily translate into a perceived need for assessment. Emotional responses may also shape how people interpret symptoms and decide when to seek medical advice (18). Previous research has described limited cancer knowledge, non-specific symptoms, inaccurate symptom attribution, and symptom trivialization as barriers to help-seeking (19–21). Informants sometimes explained symptoms as benign or considered them too minor to warrant attention. A UK qualitative study of cancer alarm symptoms similarly described normalization and concern about wasting clinicians’ time (8). Research in men with possible prostate cancer also identified symptom misinterpretation and the role of social contacts in decisions to seek help (22). These comparisons suggest that education should address how symptoms are interpreted, alongside providing cancer information. In practice, a candidate approach is to explain which persistent or changing symptoms merit discussion and whom to contact, while avoiding the implication that every urinary symptom indicates cancer. The present interviews did not test whether such education would change behavior.

Sexual concerns, anticipated judgment, and responsibilities to others appeared to make disclosure difficult for some informants (23). Previous research has shown that embarrassment, fear of illness and treatment, and expectations of the male role are barriers to help seeking (12, 24–26). The present accounts suggest that these concerns can coexist with practical pressures, such as work and family obligations, rather than operate independently. Similar interactions across personal, interpersonal, and institutional levels were described in a Nigerian prostate cancer study (21). Private opportunities to discuss urinary and sexual concerns may therefore be useful, with clinicians asking about the patient’s own priorities rather than assuming that all men endorse the same gender expectations. This is a proposed implication of the findings, not evidence that a particular communication intervention is effective.

When support is difficult to access, patients may try to manage symptoms themselves, particularly in settings with limited resources (13). Previous qualitative research has highlighted the relevance of social context to symptom appraisal and help-seeking (27, 28). Difficulties obtaining information were linked to uncertainty about post-discharge support. The contrast between requests for online advice and difficulties using digital services suggests that access depends on usability as well as availability. Previous studies have also described the information needs of patients and the importance of community support (29). Because that study involved women with different cancers, it offers contextual comparison rather than direct confirmation of the present findings. For this sample, options worth evaluating include a named contact for symptom questions, clear explanations of follow-up arrangements, and telephone or face-to-face alternatives to online communication. The interviews do not establish the effectiveness of these options or justify conclusions about the quality of the Chinese healthcare system as a whole.

A qualitative study of kidney cancer screening intentions identified low perceived risk, time constraints, and limited information as barriers, alongside social support and professional recommendations (30). Social support had more than one potential role. Family encouragement helped one informant seek assessment, yet other accounts described concealing symptoms to avoid worrying relatives or jeopardizing family responsibilities. Chinese research on help-seeking in ovarian cancer likewise identified caregiver availability, role demands, and practical burdens (31). Thus, the presence of family members should not be treated as equivalent to supportive involvement. Asking whom a patient wishes to involve, and what help that person can provide, may be more informative than marital status alone. The present data cannot determine whether married, unmarried, or widowed men experienced systematically different barriers; such comparisons would require more detailed contextual data and adequate representation.

Informants valued clear explanations, responsive advice, and reassurance during symptom management. As an interpretive lens, the HBM links symptom minimization with perceived threat, anticipated embarrassment and practical difficulty with perceived barriers, and useful advice with perceived benefits. Family encouragement may be understood as a cue to action. This mapping is an interpretation of the accounts, not a test of the model; self-efficacy and changes in later help-seeking were not established. Future longitudinal interviews could explore whether helpful encounters strengthen confidence in seeking advice and how this varies across stages of care.

### Summary

This study explored perceived barriers and facilitating factors in symptom-related help-seeking among men with genitourinary malignancies. The findings suggest that symptom-management support should consider social and cultural contexts, gender role expectations, and psychological concerns. Potential priorities include accessible professional advice and support that addresses patients’ family circumstances and perceived needs.

Several limitations should be acknowledged. The small sample included only two to four informants per cancer type, limiting exploration of tumor-specific barriers and facilitating factors. Although no new themes were reported during analysis of the overall sample, this does not establish saturation within each cancer type or age group. The findings should not be assumed to apply uniformly across cancer types or age groups. Recruitment from a single tertiary hospital further limits transferability to other settings and cultural contexts, and retrospective accounts across varying disease courses may be affected by recall bias. Family history of cancer was not systematically collected, limiting our ability to explore how these characteristics shaped symptom-related help-seeking. Future multicenter qualitative studies should recruit more informants within individual cancer types and across age groups to explore shared and distinct experiences. Longitudinal interviews could examine changes in help-seeking across treatment stages.

## Data Availability

The original contributions presented in the study are included in the article/supplementary material. Further inquiries can be directed to the corresponding author.
